# Examining educational attainment and allostatic load in non-Hispanic Black women

**DOI:** 10.1186/s12905-022-01641-0

**Published:** 2022-03-17

**Authors:** Brittany Marie Williams, Christian Laurent, Rishab Chawla, Justin Xavier Moore

**Affiliations:** 1 Department of Educational Leadership and Higher Education, St. Cloud State University, St. Cloud, MN USA; 2 Cancer Prevention, Control, and Population Health Program, Department of Medicine, Georgia Cancer Center, Augusta University, 1410 Laney Walker Blvd CN-2135, Augusta, GA 30912 USA; 3 Institute of Preventive and Public Health, Medical College of Georgia, Augusta University, Augusta, GA USA

**Keywords:** Allostatic load, Cumulative stress, Education and health, Black women’s health, Health disparities

## Abstract

**Background:**

Research suggests that non-Hispanic Black (henceforth, Black) women and people with lower educational attainment have higher levels of allostatic load (AL). This study sought to determine the association between educational attainment and AL among a large sample of Black women.

**Methods:**

We analyzed data among 4177 Black women from the National Health and Nutrition Examination Survey years 1999–2018. AL score was defined as the total for abnormal measures of eight biomarkers. We further categorized participants with AL score greater than or equal to 4 as having high AL. We calculated mean estimates of total allostatic load scores using generalized linear models. We performed modified Poisson Regression models with robust variance estimation to estimate prevalence ratios (PRs) of high allostatic load and their associated 95% confidence intervals (CIs) by educational attainment.

**Results:**

Black women with a college degree or higher had the lowest prevalence of high allostatic load (31.8% vs. 42.7%, 36.3%, 36.6%), and age adjusted mean allostatic load scores (mean = 1.90 vs. mean = 2.34, mean = 1.99, mean = 2.05) when compared to Black women with less than a high school diploma, high school diploma or GED, and some college or associates degree respectively. Even after accounting for age, poverty-to-income ratio, smoking, congestive heart failure, and heart attack, Black college graduates had an 14.3% lower prevalence of high allostatic load (PR = 0.857, 95% CI 0.839–0.876) when compared to Black women with lower educational attainment.

**Conclusions:**

Black women with a baccalaureate degree or higher educational attainment had lower allostatic load compared to Black women with less than a high school education. This finding further confirms higher education is a social determinant of health. Future research should explore differences in AL by more granular degree types.

**Supplementary Information:**

The online version contains supplementary material available at 10.1186/s12905-022-01641-0.

## Introduction

In 1988, Joseph Eyer and Pete Sterling coined the term *Allostasis* to refer to the process through which the body achieves homeostasis despite changing environmental conditions and stressors [1–3]. Whereas homeostasis refers to the body’s inherent tendency to return to its natural resting point, allostasis typically connotes a response to specific psychosocial stressors. Thus, allostatic load (AL) can be thought of as chronic stress. Chronic stress, when sustained over time, may cause one’s body to lose the ability to downregulate immunologic and neuroendocrine mechanisms. The resulting impact of this inability to return to one’s resting point is the manifestation of elevated biomarkers. A plausible mechanism for the pathogenesis of allostasis is Hans Selye’s General Adaptation Syndrome, which states that compounding stress manifests biologically as alarm, resistance, and exhaustion [4]. Following prolonged activation of the alarm phase, the increased hormones eventually lower the body’s resistance to maintain baseline allostasis. When the stress exposure is chronic, the body can no longer maintain a proper balance, homeostasis [5]. The negative feedback loop is dysregulated resulting in exhaustion that leads to an increased AL [2].

Higher AL has been associated with a range of physical and cognitive diseases, such as cardiovascular disease and memory decline [2]. For instance, a recent systematic review of 267 studies reported that higher AL was associated with poorer health outcomes [6]. Notably, the effects of AL vary dramatically by social identities such as race, ethnicity, age, gender, and sexual orientations [7]. Regardless of the time between 1988 and 2018, Moore et al. reported that non-Hispanic Black (henceforth, Black) people had up to a 29.2% increased risk of high AL compared to their non-Hispanic white counterparts [7]. Even when accounting for socioeconomic status, Black women had the highest allostatic load when compared to any other race-gender specific subgroup including white women and men, and Black men [8].

### Purpose

The purpose of this study was to explore whether higher education is associated with higher allostatic load among a sample of Black women using National Health and Nutrition Examination Survey (NHANES) reported data. We examined a 19-year timespan of NHANES data to consider the effect of educational attainment on AL among Black women. As in prior studies, AL was operationalized in biological terms as the sum of various cardiovascular and metabolic biomarkers and thus allowed for a direct quantitative appraisal of differences in AL burden and prevalence, both crude and adjusted for age, poverty level, or chronic disease status.

### Understanding AL and Black women

Geronimus et al. hypothesized systemic stress is a form of physical “weathering” due to the sustained impact high AL has on Black people’s health outcomes [9]. When considered alongside Black American’s ongoing endurance of and genealogical lineage to the brutalities of the trans-Atlantic slave trade and the 1960s Civil Rights movement to contemporary disparities in COVID-19 cases, one can infer that these inter- and cross-generational stressors further exacerbate already exceedingly high physiological deteriorations for the Black community., Black women, then, continue to experience multiple forms of multifactorial and layered discrimination (by race, sex, and class) [10]. Indeed, Kimberlé Crenshaw named these nexuses of layered discrimination as a problem of intersectionality in 1991. Since Black women endure and navigate the world with multiple intersectional marginalized identities, one can infer that their lived experiences may synergistically manifest on their bodies and prove more deleterious than one individual source of discrimination.

Moore et al. observed that age-adjusted mean AL scores were highest among Black women at the end of the 30-year period [7]. However, when considering within-group differences among Black women, appraisal of stressors such as racial discrimination can vary when considered with socioeconomic status. Allen et al. observed higher levels of circulating epinephrine and waist circumference in Black women with lower education attainment as compared to those with higher education attainment while adjusting for levels of self-reported racial discrimination [11]. Similarly, educational attainment is rarely isolated from other indicators of socioeconomic status (SES), making studies that specifically examine connections among Black women’s educational attainment and allostatic load sparse. Among those available, a longitudinal study suggests that both Black women and individuals with lower educational attainment have higher AL, but these factors were studied independently of each other [12].

A cross-sectional study including individuals from NHANES and US Census data found that lower education level and being either Black or female was associated with a higher AL, but the intersection of the three variables was not studied [13]. This problem of single axis identity analysis underscores why intersectional research on Black women (the nexus of subordinated race and gender identities) is necessary. Correspondingly, an analysis of data from the National Longitudinal Study of Adolescent to Adult Health found that completion of higher education was no more or less protective for AL in Black women [14]. While methodological and technical limitations introduce difficulty in correlating perceived stress with adverse AL, there is an urgent need to explore the impact of social stressors on Black women in association with their educational achievement. Such studies will require researchers to better disaggregate Black women as a distinct demographic category and population deserving of examination [15–18].

Furthermore, even when controlling for age, poverty status, and underlying chronic diseases, Black women without a college degree are found to have higher AL prevalence. This finding further confirms existing data indicating Black women endure unique daily stressors that are not fully explained by underlying structural determinants and necessitate acknowledgment of intersectional interpersonal and institutional factors. For example, the Black Women’s Health Study (BWHS) found that workplace discrimination was associated with a 30% increase in breast cancer risk among Black women [19]. A follow-up study from the same participant pool identified a positive association between both personally experienced and institutionalized racism with incidence of hypertension [20].

Ultimately, there is an urgent need to better understand how education might mitigate high levels of AL given the layered discrimination Black women experience [21]. Unpacking these within group differences can help nuance data attributed to Black women broadly like the superwoman schema, a culturally-specific framework characterizing a psychosocial response to stress among Black women. The superwoman schema plays an important role in the upregulation and downregulation of AL [11]. While it is reported that superwoman schema reduced AL through the need to present an image of strength, the simultaneous intense motivation to succeed and feelings of obligation to help others increased AL for Black women [11]. Given the success pressures and filial obligations associated with the educational attainment, the findings from this study will play an important role in building upon existing research by illuminating the within-group differences among Black women’s AL by educational attainment.

## Methods

### Study design and participants

We performed statistical analysis with cross-section data from NHANES between 1999 and 2018. NHANES is a nationally representative sample of US adults, where persons aged 60 and older, Latinos and Blacks are oversampled, and weighted analysis generates generalizable population estimates [22]. Since 1959, The National Center for Health Statistics (NCHS) of the Centers for Disease Control and Prevention (CDC) has collected, analyzed, and disseminated data on the health status of US residents [23]. Using stratified multi-stage probability sampling, NHANES enrolls a nationally representative sample of about 5000 non-institutionalized civilians annually. Those selected to participate are initially interviewed in their homes by trained NHANES personnel, who administer questionnaires using computer assisted technology for standardization. One to two weeks after the household interview, participants are requested to visit a Mobile Examination Center (MEC) to complete additional interviews, examinations, and laboratory assessments. NHANES collects demographic, socioeconomic, dietary, health-related questionnaires, and includes clinical measures of blood pressure, fasting blood glucose, triglycerides, and HDL cholesterol, in addition to self-reported medication use for health conditions. We used NHANES years with consistent data on the component variables of allostatic load, i.e., 1999 through 2018 [24]. We excluded participants younger than 18 or pregnant from study. This analysis included all Black women aged 18 and older with available data on allostatic load components, and no missing information on educational attainment, and household income. The resulting 4,177 participants over the 20-year study period served as our analytic population.

### Ethics and consent to participate/informed consent

This study was considered exempt by the Institutional Review Boards of Augusta University and St. Cloud State University due to use of existing secondary data that are publicly available and non-identifiable. Original NHANES investigators maintained informed consent for all participants surveyed. Health information collected in the NHANES is kept in strictest confidence. During the informed consent process, survey participants are assured that data collected will be used only for research purposes and will not be disclosed or released to others without the consent of the individual or the establishment in accordance with section 308(d) of the Public Health Service Act (42 U.S.C. 242m).

### Outcome variable: allostatic load

Definitions of AL vary though most incorporate biomarker measures from three categories of physiologic functioning: cardiovascular, metabolic, and immune systems [25]. As there is no consensus definition, we elected to define AL using the Geronimus et al. (2006) and Mays et al. (2018) taxonomies [9, 26]. To determine the high-risk thresholds for each AL component, we examined the distribution of each component among Black women for whom complete biomarker data was available in the NHANES study sample (*N* = 4177 participants). High-risk thresholds were determined as either (a) above the 75th percentile for body mass index (BMI), diastolic blood pressure (DBP), systolic blood pressure (SBP), glycated hemoglobin, total cholesterol, and serum triglycerides; or (b) below the 25th percentile for serum albumin and serum creatinine. Each participant was scored as either 1 (high risk) or 0 (low risk) based on the following cutoffs for each component (See Additional file 1: Table S1): (1) serum albumin ≤ 4.03 g/dL, (2) BMI ≥ 32.1 kg/m^2^, (3) creatinine ≤ 81 μmol/L, (4) DBP ≥ 81.00 mmHg, (5) glycohemoglobin % ≥ 5.86, (6) SBP ≥ 135.00, (7) total cholesterol ≥ 212.00 mg/dL, and (8) serum triglycerides ≥ 145.00 mg/dL. We calculated a total AL score, ranging from 0 to 8, by summing the individual components based on the high-risk thresholds. We further categorized participants with AL scores greater than or equal to 4 as having high AL [25, 26].

### Independent variable and other variables of interest

Our independent variable of interest was educational attainment. We categorized NHANES educational attainment into four groups: (1) less than a high school education; (2) high school graduate, GED, or equivalent; (3) some college; and (4) college graduate or above. We were unable to differentiate by degree types due to the NHANES data collection categorization.

We also included other variables typically connected to our area of inquiry. NHANES calculated income relative to federal poverty line (PIR) as the ratio of total family income to poverty threshold values. Persons who reported having had no income were assigned a zero value for PIR. PIR values less than 1 are considered below the official poverty line, whereas PIR values greater than 1 are above the poverty level, and values near 5 are considered very high income. We evaluated personal characteristics that may influence AL score, including age at first menarche, total number of pregnancies, parity, depression, smoking status, waist circumference, and comorbidities. Age at first menarche was defined by self-reported response to “How old were you when you had your first menstrual period?” Total number of pregnancies was determined by self-reported response to “How many times have you been pregnant? Be sure to count all pregnancies including live births, miscarriages, stillbirths, tubal pregnancies or abortions.” We defined parity as the total number of pregnancies resulting in live births. Twins and multiple births counted as a single delivery. During 1999–2004 NHANES survey periods, diagnostic modules were administered that addressed diagnoses of depressive disorders within the past 12 months. Participants were diagnosed with depression according to definitions and criteria of the tenth revision of the International Classification of Diseases (ICD-10) and the fourth edition of the American Psychiatric Association’s Diagnostic and Statistical Manual of Mental Disorders (DSM-IV).

However, for years 2005 through 2018, NHANES obtained depressive disorder through the Patient Health Questionnaire-9 (PHQ-9) [27–29]. The PHQ-9 was similarly administered through face-to-face MEC and assessed depressive symptoms over the past two weeks [27–29]. Participants indicated on a scale from 0 to 3, the frequency with which they experienced the following symptoms: (1) inability to feel pleasure, (2) depressed mood, (3) sleep disturbance, (4) fatigue, (5) appetite changes, (6) low self-esteem, (7) concentration problems, (8) psychomotor disturbances, and (9) suicidal ideation [27–29]. PHQ-9 scores range from 0 to 27 with scores ≥ 10 representing clinically significant depressive symptoms [29]. Using these definitions, we categorized participants in NHANES 1999–2004 as living with depressive disorder if diagnosed based on ICD-10 and DSM-IV, and participants within NHANES 2005–2018 as living with depressive disorder if having a PHQ-9 score ≥ 10. Smoking status was categorized as a discrete, mutually exclusive, three-level variable. Participants who had smoked fewer than 100 cigarettes in their lifetime were categorized as never smokers; participants who had smoked at least 100 cigarettes in their lifetime but were not current smokers were categorized as past smokers. Participants who had smoked at least 100 cigarettes in their lifetime and continued to smoke were categorized as current smokers. NHANES purposefully collected high quality body measurement data participants including waist circumference (in centimeters). We examined these variables as continuous variables. NHANES questioned “Has a doctor or other health professional ever told you that you had … (cancer, angina, congestive heart failure, or heart attack?” Thus, we included any self-reported response to a physician-diagnosed history of cancer, angina, congestive heart failure, or heart attack as comorbidities. These comorbidities were examined as discrete binary variables (i.e., yes or no for each condition).

### Statistical analysis

We performed analyses using NHANES generated sampling statistical strata, clusters, and weights as designated and described in detail within the NHANES methodology handbook [22]. We reported categorical variables as weighted row percentages, continuous variables as mean and associated standard errors (SEs), and non-normal continuous variables as median and associated first (Q1) and third quartiles (Q3). The primary outcomes of interest were: (1) dichotomous AL (i.e., high or low) and AL total scores (ranging from 0 to 8). We performed several sequential adjusted modified Poisson regression models for estimating relative prevalence of high AL by educational attainment [30–32]. We adjusted for potential confounders including: age groups, total number of pregnancies, age at menarche, PIR, depressive disorder, smoking status, any history of cancer, congestive heart failure, and heart attack [5, 7, 33]. We estimated the variance for point estimates within the modified Poisson regression utilizing the delete-1 jackknife (resampling) method [30–32]. Next, we conducted weighted generalized linear models to examine the association between the AL total score and educational levels adjusted for age [34, 35]. We conducted sensitivity analysis to examine whether the association between educational attainment and prevalence of high allostatic load were modified by age groups. Estimates derived from log-binomial are presented as prevalence ratios (PRs) and associated 95% confidence intervals (CIs), and estimates derived from generalized linear regression models are presented as mean allostatic load estimates and associated 95% CIs. We considered p values less than 0.05 as statistically significant. We used SAS software version 9.4 (copyright © 2013 SAS Institute Inc., Cary, NC, USA) for all analyses.

## Results

### Characteristics of the study population

We analyzed data among 4177 Black women within NHANES years 1999 through 2018, representing an estimated 9,494,904 non-institutionalized US (Table 1). The majority of Black women reported attaining some college or an associate’s degree (35.7%, weighted); followed by those with a high school diploma or GED (24.5%, weighted), less than a high school diploma (21.9%, weighted), and a college degree or higher (17.8%, weighted). Among all Black women, the mean age was 43.6 years (SE = 0.3), and nearly one in four women were aged 18 to 29 (weighted % = 24.3, SE = 0.8). Black women college graduates on average had higher mean PIR (3.43, SE = 0.07) when compared with Black women with other educational attainments. Black women college graduates had lower total number of pregnancies (median = 2.03, Q1–Q3 = 1.10–3.30), lower parity (median = 1.37, Q1–Q3 = 0.61–2.19), and lower proportion of depressive disorder (weighted % = 6.6, SE = 1.04) when compared to Black women with lower educational attainment levels. Black women college graduates also had younger mean age at menarche (12.50 years, SE = 0.08) when compared with Black women with less than high school education (12.86 years, SE = 0.07). Black women college graduates had lower mean waist circumference (98.6 cm, SE = 0.8), lower proportion of current smoking status (weighted % = 5.5, SE = 0.9) and lower proportions of angina (weighted % = 0.9, SE = 0.3), congestive heart failure (weighted % = 1.4, SE = 0.4), and heart attack (weighted % 1.6, SE = 0.5) when compared with Black women with other educational levels.

**Table 1:** Demographic characteristics, personal health, and medical conditions by educational status, among 4177 Black women, an estimated 9,494,904 US residents from the National Health and Examination Survey (NHANES) 1999–2018

Characteristic	Less than high school	High school/GED	Some college or associates degree	College graduate or higher	All
Participants (N)	1020	1089	1397	671	4177
Estimated N^a^ (Weighted %)	2,082,665 (21.9)	2,330,386 (24.5)	3,391,159 (35.7)	1,690,695 (17.8)	9,494,904
Allostatic load total score, mean (SE)^b,c^	2.34 (0.05)	1.99 (0.05)	2.05 (0.05)	1.90 (0.07)	2.07 (0.03)
High allostatic load, % (SE)^d,e^	42.7 (1.5)	36.3 (1.6)	36.6 (1.4)	31.8 (2.0)	37.0 (0.9)
Age in years, mean (SE)^c^	48.2 (0.6)	41.9 (0.6)	41.7 (0.5)	44.3 (0.6)	43.6 (0.3)
*Age group, years*, % (SE)^e^					
18–29	20.5 (1.3)	30.8 (1.6)	26.8 (1.4)	15.0 (1.4)	24.3 (0.8)
30–39	17.1 (1.2)	17.7 (1.3)	21.3 (1.2)	24.0 (1.8)	20.0 (0.7)
40–49	16.7 (1.4)	16.2 (1.2)	22.8 (1.4)	27.9 (2.1)	20.7 (0.7)
50–59	14.6 (1.4)	16.4 (1.3)	15.3 (0.9)	18.9 (1.4)	16.1 (0.7)
60–69	13.5 (1.0)	12.0 (0.9)	8.8 (0.6)	8.5 (0.9)	10.6 (0.5)
70+	17.7 (1.2)	6.8 (0.7)	5.0 (0.5)	5.7 (0.8)	8.4 (0.5)
Total number of pregnancies, median (Q1, Q3)	3.39 (1.90, 5.17)	2.66 (1.48, 4.04)	2.45 (1.43, 3.75)	2.03 (1.10, 3.30)	2.31 (1.35, 3.55)
Parity, median (Q1, Q3)	2.55 (1.40, 4.13)	1.92 (0.99, 2.94)	1.59 (0.82, 2.54)	1.37 (0.61, 2.19)	1.73 (1.05, 2.68)
Age at menarche, mean (SE)	12.86 (0.07)	12.64 (0.06)	12.44 (0.05)	12.50 (0.08)	12.69 (0.02)
Income relative to federal poverty line, mean (SE)^c^	1.43 (0.05)	1.77 (0.06)	2.27 (0.05)	3.43 (0.07)	2.17 (0.05)
Depressive disorder, % (SE)	18.9 (1.85)	9.4 (1.35)	11.3 (1.01)	6.6 (1.04)	7.6 (0.25)
Waist circumference in cm, mean (SE)^c^	100.1 (0.5)	99.0 (0.7)	100.6 (0.5)	98.6 (0.8)	99.7 (0.3)
Current smoker status, % (SE)^e^	29.8 (1.6)	22.3 (1.6)	18.6 (1.2)	5.5 (0.9)	19.6 (0.8)
Any cancer history, % (SE)^e,f^	4.4 (0.7)	4.2 (0.6)	4.1 (0.5)	4.2 (0.7)	4.2 (0.3)
Angina, % (SE)^e^	2.3 (0.5)	1.6 (0.4)	2.4 (0.4)	0.9 (0.3)	1.9 (0.2)
Ever congestive heart failure, % (SE)^e^	5.1 (0.7)	2.5 (0.5)	2.1 (0.4)	1.4 (0.4)	2.7 (0.3)
Ever heart attack, % (SE)^e^	3.6 (0.6)	2.1 (0.4)	1.8 (0.3)	1.6 (0.5)	2.2 (0.2)

^a^Estimated using sampling weights from National Health and Nutrition Examination Survey (NHANES)

^b^ Allostatic load total score was calculated as sum total of components based on high-risk thresholds: albumin, BMI, creatinine clearance, diastolic blood pressure, glycated hemoglobin, systolic blood pressure, total cholesterol, triglycerides. Score ranges from 0 to 8

^c^Presented as weighted mean (standard error) for continuous variables

^d^High allostatic load defined as total allostatic load score greater than or equal to 4

^e^Presented as weighted column proportion (standard error)

^f^Defined as self-reported response to ever being diagnosed by a doctor or health professional of any cancer or malignancy

### High allostatic load

Black women college graduates had the lowest proportion of high allostatic load (weighted % = 31.8) when compared to other Black women with lower educational attainment. In the unadjusted model, Black women with higher education were less likely to have high allostatic load when compared to Black women with less than high school education; a 25.4% lower prevalence for college graduates (Table 2: crude PR = 0.746, 95% CI 0.738–0.753), a 14.2% lower prevalence for those with some college or Associate’s degree (crude PR = 0.858, 95% CI 0.857–0.859), and a 15.0% lower prevalence for those with a high school diploma or GED (crude PR = 0.850, 95% CI 0.849–0.850). In further age-adjusted models, Black women with a higher education were less likely to have high allostatic load when compared with Black women with less than a high school education; a 5.4% lower prevalence for those with high school diploma or GED (model 1 PR = 0.946, 95% CI 0.937–0.954), an 4.1% lower prevalence for those with some college or Associate’s degree (model 1 PR = 0.959, 95% CI 0.952–0.967), and a 24.2% lower prevalence for those with a college degree (model 1 PR = 0.758, 95% CI 0.750–0.767). After further adjustments for other possible confounders including total number of pregnancies, age at menarche, poverty to income ratio, depressive disorder, smoking status, and comorbidities, we observed that Black women with higher educational attainment were less likely to have high allostatic load when compared to Black women with less than high school education. One of the strongest effects was observed among Black women college graduates compared with Black women with less than high school education (model 3 PR = 0.857, 95% CI 0.839–0.876).

**Table 2:** Association between education and high allostatic load presented as prevalence ratios (PRs) and associated 95% confidence intervals (CIs), among 4177 participants, using NHANES weighting an estimated 9,494,904 US Black women

	No. (weighted %)^a^	Prevalence ratios (95% confidence interval)			
		Crude	Model 1^b^	Model 2^c^	Model 3^d^
*Education level*					
Less than high school (Referent)	443 (25.3)	1.000 (Referent)	1.000 (Referent)	1.000 (Referent)	1.000 (Referent)
High school/GED	393 (24.1)	0.850 (0.849–0.850)	0.946 (0.937–0.954)	0.932 (0.925–0.940)	0.926 (0.920–0.933)
Some college or associates degree	546 (35.3)	0.858 (0.857–0.859)	0.959 (0.952–0.967)	1.005 (0.993–1.017)	1.027 (1.013–1.041)
College graduate or higher	226 (15.3)	0.746 (0.738–0.753)	0.758 (0.750–0.767)	0.835 (0.819–0.851)	0.857 (0.839–0.876)

Prevalence ratios for high allostatic load are estimated using modified Poisson regression with robust variance estimation and accounting for NHANES weighting. Confidence intervals estimated using delete-1 jackknife method accounting for complex statistical weighting, cluster, and strata

^a^Number of participants with high allostatic load per stratum (weighted stratum proportion with high allostatic load)

^b^Model 1: Adjusted for age only

^c^Model 2: Additionally adjusted for total number of pregnancies, age at menarche, and poverty to income ratio,

^d^Model 3: Additionally adjusted for depressive disorder, smoker status, ever congestive heart failure, and ever heart attack

### High allostatic load stratified by age groups

We performed additional multivariable modeling to examine the relationship between educational attainment and prevalence of high allostatic load, stratified by age groups (i.e., 18–29, 30–39, 40–49, and 50 and older). We present these results in Additional file 2: Table S2. We observed that particularly for those aged between 18 and 29, college degree educational attainment was associated with greater prevalence for higher allostatic load in crude (PR = 1.141, 95% CI 1.124–1.158) and after adjustments for personal level characteristics including number of pregnancies, age at menarche, and poverty-to-income ratio (PR = 1.152, 95% CI 1.124–1.180) when compared to those with less than high school education. However, this increased prevalence attenuated after further adjustments for other health conditions and those with college degree or higher were at a reduced prevalence of high allostatic load (PR = 0.641, 95% CI 0.623–0.660). In general, for women aged 30 through 49, the relationships between educational attainment and allostatic load mirrored our main results, with greater educational attainment usually associated with lower prevalence for high allostatic load. However, among those aged 50 and older, the association between higher educational attainment and reduced prevalence of high allostatic load was not as pronounced in crude (PR = 0.841, 95% CI 0.838–0.846), model 1 adjusted (PR = 0.968, 95% CI 0.963–0.973), and model 2 adjusted (PR = 1.021, 95% CI 1.014–1.027) models.

### Mean estimates for allostatic load scores

**Fig. 1:**
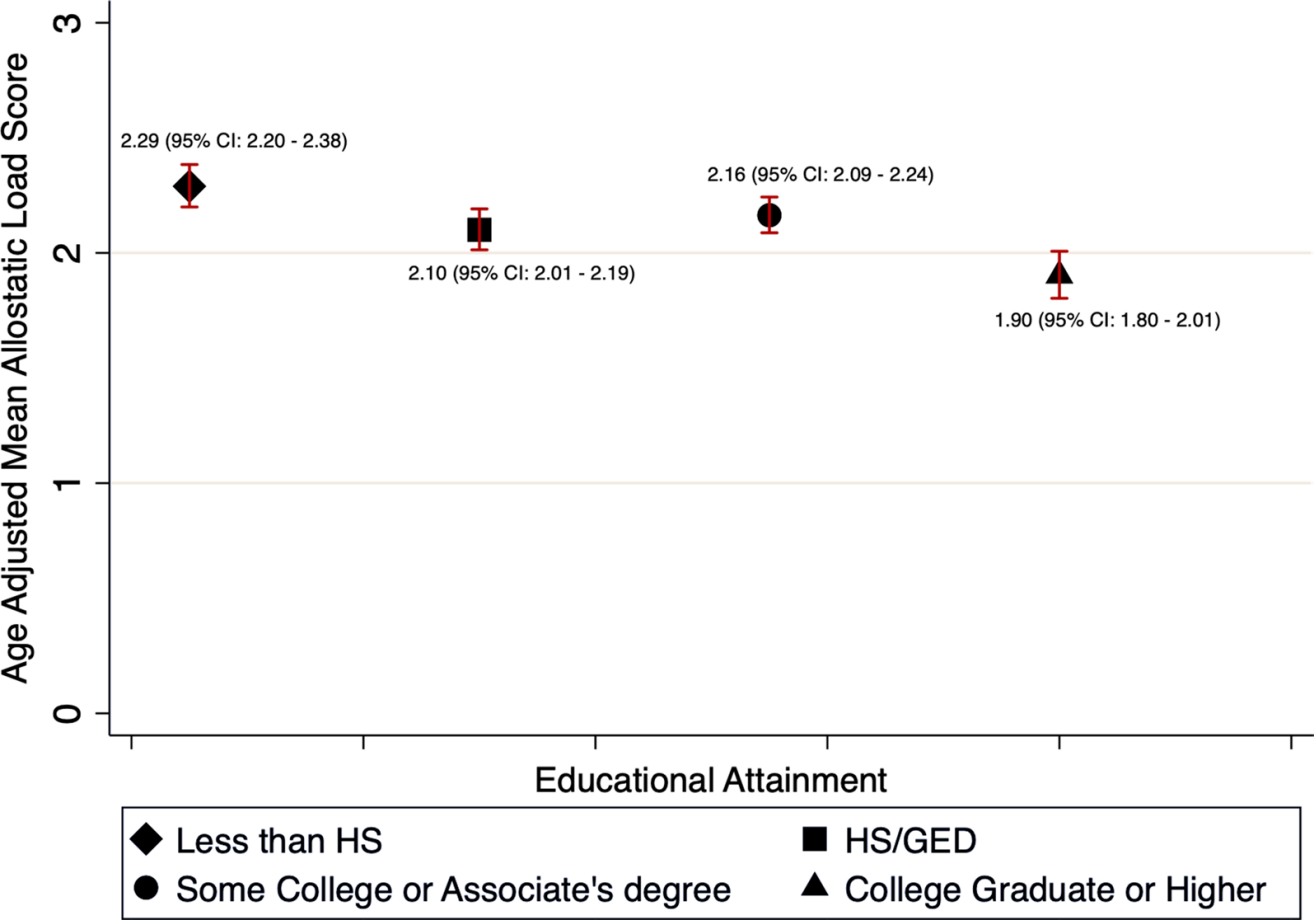
Age-adjusted mean allostatic load scores by educational attainment among 4177 participants, using NHANES weighting an estimated 9,494,904 US Black Women. National Health and Examination Survey (NHANES) 1999–2018

We additionally examined the relationship between educational attainment and age-adjusted mean scores for allostatic load among Black women. We observed that Black women with a college degree or higher had lower age-adjusted mean allostatic load scores (Fig. 1: mean AL = 1.90, 95% CI 1.80–2.01), when compared to all other lower educational attainment.

## Discussion

In this population-based study among non-institutionalized Black women, we observed that Black women with higher educational attainment, a college degree or higher, had both the lowest prevalence for high allostatic load and lower age-adjusted mean levels of allostatic load scores when compared to Black women with lower educational attainment. Even after accounting for several possible socio-economic and health related confounders, college-educated Black women had more than an 18% lower prevalence of high allostatic load compared to Black women with less than high school education. Conversely, in the fully-adjusted models, Black women with some college completion or associate degree were observed to have consistently higher allostatic load compared to college-educated Black women. Our latter finding raises some concerns regarding the associated stress of lower-level college completion and dropout status. The culmination of these findings suggests there is an ongoing connection between education and AL for Black women.

Prior research indicates prolonged activation of the body’s natural stress response results in high AL [1]. Much research has focused on disease states and mortality risk caused by AL while only secondarily examining the negative impact of high AL on daily life and how other factors like education are negatively correlated with AL [2, 8, 12]. In this study, we further corroborated the relationship between education attainment and AL levels, thereby further implicating education as a social determinant of health. Studies often correlate demographic characteristics such as age, race, gender, and poverty status with AL [9]. By (re)considering the intracommunal (within group) differences among Black women, our findings underscore how education attainment levels might explain within group disparities of AL levels. Though not a primary focus in our examination, researchers agree racial discrimination plays an important role in the relationship between racial identity and AL [5, 11, 33, 36–38]. Racial minorities, specifically Black women, are exposed to higher levels of racial discrimination that may in turn explain the prolonged activation of the body’s natural stress response [5, 11, 33]. Specifically, Black women tend to have higher AL than their white women and Black men counterparts [7], despite the association of lower AL level with reported incidents of very-high institution-specific racial discrimination in a prior study [33]. This increased racial stress exposure may lead to greater experiences of anger, and poorer quality of life which in turn may lead to higher levels of cumulative stress and AL [35–38]. However, there is evidence suggesting education attainment has some influence on these realities [5, 11, 33].

Much of the existing literature suggests lower educational attainment increases mortality and reduces longevity [39–42]. In fact, Black women with the highest educational attainment who also self-reported high racial discrimination rates were also, still, associated with lower AL [5]. These disparate findings clarify that Black women endure unique daily stressors that are not always fully explained by underlying structural determinants and signal that education has some role in the mitigation of AL levels. Indeed, our findings further confirm existing research that lower education (despite levels of self-reported racial discrimination) is associated with higher AL [5]. Even when controlling for age, poverty status, and underlying chronic diseases, we found that Black women without a college degree had higher AL thus underscoring the need for more granular exploration of the relationship with education on AL. It is plausible, then, to conclude that coping, higher health literacy and access, and greater positive social support all of which are connected to college outcomes further underscore education as a social determinant of health for Black women [21].

Indeed, Link and Phelan’s (1995) fundamental cause theory requires that we consider how educational attainment is deeply intertwined with multiple disease outcomes, longevity, and disease impacts [39, 43]. AL disparities, which we link to education attainment, is similarly correlated with several deleterious health outcomes for Black women such as a poorer breast cancer survival [44], greater adverse pregnancy outcomes [45], and higher incidence of cardiovascular disease [46]. Since education influences health and longevity even when the mechanisms linking it to health change, our findings on AL and education attainment reveal how the college context and outcomes of a college degree are especially critical in improving Black women’s overall health [39, 43]. Greater educational attainment may in turn provide multiple pathways to mitigate overall risk of mortality and increase longevity for Black women [39]. As research continues to highlight that higher education attainment is positively associated with better health outcomes [40, 41] and lower education attainment is associated with shorter life expectancy and adverse outcomes [41], understanding more of the within group disparities along these issues can help with better targeting health problems at their source. Leveraging a socio-ecological framework, for instance, that centers on the interplay through personal (e.g., personal coping skills and health resources), interpersonal (e.g., colleagues with higher education and resources), and community (e.g., neighborhood quality and access to care), and societal factors can improve within group AL disparities among Black women [39, 43].

Taken together, the disparities Black women endure at the nexus of education attainment and AL align with the broader social context as U.S. leaders often propagate educational attainment as a prerequisite for prosperity [47]. The culmination of our findings as presented when considered alongside the broader scholarly canon offer additional support for leveraging higher educational attainment to improve societal health generally and the health for Black women specifically. While the findings from this study have broad implications, the results should be viewed in light of certain strengths and limitations. NHANES is a nationally representative, standardized survey on various health related conditions. As a result, the findings are generalizable to non-institutionalized US citizens and have great external validity. This study focuses on non-Hispanic Black women, and thus the results are limited to this population and future studies should explore the relationship between education and AL in other race-ethnicity-gender populations.

Due to the cross-sectional study design, we are unable to examine causal associations between education between education and AL. Participants were surveyed on a variety of health-related conditions and behaviors, and as a result is likely that there are minimal misclassification biases among our primary exposure, educational attainment. Further, there is no consensus on measurement of AL using population-based data. Thus, we may have underestimated measures of AL by using most available components over the study period. NHANES did not collect granular data on educational attainment (i.e., college graduates and more were all categorized together). As a result, we were unable to examine the association between AL and specific higher educational attainment. Future studies within a large prospective cohort have the potential to further explore the associations between race, education, and development of allostatic load. Moreover, our intracommunal differences may reveal a different story when the analysis considers populations beyond the United States alone. Future studies should examine other AL for Black women outside the U.S. as well as factors associated with higher educational attainment such as increased self-advocacy skills to further delineate what factors, beyond increased socioeconomic status attributed to higher education, mediate differences in AL.

## Conclusion

Prior research indicates that Black women generally have higher levels of AL compared with other race-gender groups, including White women, Black men, and white men. However, the findings of the present study further elucidate that higher education, specifically a college degree or higher, is associated with lower AL among Black women. While our findings delineate Black women with at least a bachelor’s degree hold the lowest prevalence of AL when compared to Black women with lower educational attainment, future research is needed to further disentangle what kinds of education and degrees are most associated with mitigating high AL and to examine factors that may mediate the relationship between education and allostatic load. Education remains a significant indicator of long-term health, and reducing AL disparities by educational attainment may require more holistic efforts between public policy makers, health care workers, and educators alike.

## Supplementary Information

**Additional file 1: Supplemental Table 1.** Weighted distribution of allostatic load components comparing subsample of non-Hispanic Black women vs. entire NHANES sample, 1999 through 2018.

**Additional file 2: Supplemental Table 2.** Association Between Education and High Allostatic Load Stratified by Age Groups, Presented as Prevalence Ratios (PRs) and Associated 95% Confidence Intervals (CIs), among 4,177 participants, using NHANES weighting an estimated 9,494,904 US Black Women.

## Data Availability

The data for this study are already publicly available through the National Center for Health Statistics (NCHS), National Health and Nutrition Examination Survey (NHANES) website: https://www.cdc.gov/nchs/nhanes/index.htm.

## Abbreviations

AL: Allostatic load
BMI: Body mass index
BWHS: Black Women’s Health Study
CDC: Centers for Disease Control and Prevention
CI: Confidence interval
DBP: Diastolic blood pressure
GED: General Education Development
N or No.: Number of participants
NCHS: National Center for Health Statistics
NHANES: The National Health and Nutrition Examination Survey
NH: Non-Hispanic
PIR: Poverty income ratio
PR: Prevalence ratio
SBP: Systolic blood pressure
SE: Standard error
US: United States
