# Supplementary material for: Examining educational attainment and allostatic load in non-Hispanic Black women
Source: BMC Womens Health. 2022 Mar 17;22:75. doi: 10.1186/s12905-022-01641-0 (PMC8928016; doi:10.1186/s12905-022-01641-0)
Supplement: Supplementary file 1 — Additional file 1: Supplemental Table 1. Weighted distribution of allostatic load components comparing subsample of non-Hispanic Black women vs. entire NHANES sample, 1999 through 2018. [file 12905_2022_1641_MOESM1_ESM.docx]

| Supplemental Table 1. Weighted distribution of allostatic load components comparing subsample of non-Hispanic Black women vs. entire NHANES sample, 1999 through 2018. | | | | |
| --- | --- | --- | --- | --- |
|  | **Mean (SE)** | **Min** | **Median (Q1, Q3)** | **Max** |
| **Allostatic Load Component** |  |  |  |  |
| **Serum albumin (g/dL)** |  |  |  |  |
| non-Hispanic Black women (N = 4,177) | 4.06 (0.007) | 2.50 | 4.01 (3.81, 4.21) | 5.10 |
| Entire NHANES Sample (N = 41,169) | 4.30 (0.004) | 1.90 | 4.25 (4.03, 4.47) | 5.70 |
| **Body Mass Index (kg/m^2^)** |  |  |  |  |
| non-Hispanic Black women (N = 4,177) | 31.6 (0.14) | 15.1 | 30.4 (25.6, 36.1) | 84.9 |
| Entire NHANES Sample (N = 41,169) | 28.6 (0.066) | 13.2 | 27.5 (23.9, 32.0) | 130.2 |
| **Creatinine (mmol/L)** |  |  |  |  |
| non-Hispanic Black women (N = 4,177) | 74 (0.84) | 26.5 | 70 (61, 79) | 1043 |
| Entire NHANES Sample (N = 41,169) | 78 (0.21) | 18 | 74 (62, 88) | 1574 |
| **Diastolic Blood Pressure (mmHg)** |  |  |  |  |
| non-Hispanic Black women (N = 4,177) | 71 (0.27) | 18 | 70 (62, 78) | 124 |
| Entire NHANES Sample (N = 41,169) | 71 (0.14) | 4 | 70 (63, 78) | 134 |
| **Glycohemoglobin^1^ (%)** |  |  |  |  |
| non-Hispanic Black women (N = 4,177) | 5.75 (0.018) | 3.70 | 5.45 (5.17, 5.84) | 17.5 |
| Entire NHANES Sample (N = 41,169) | 5.55 (0.008) | 2.00 | 5.33 (5.08, 5.63) | 18.0 |
| **Systolic Blood Pressure (mmHg)** |  |  |  |  |
| non-Hispanic Black women (N = 4,177) | 124 (0.37) | 74 | 119 (109, 134) | 238 |
| Entire NHANES Sample (N = 41,169) | 123 (0.17) | 66 | 119 (110, 130) | 270 |
| **Total cholesterol (mg/dL)** |  |  |  |  |
| non-Hispanic Black women (N = 4,177) | 190 (0.84) | 82 | 185 (161, 213) | 463 |
| Entire NHANES Sample (N = 41,169) | 195 (0.39) | 59 | 191 (165, 220) | 727 |
| **Serum triglycerides (mg/dL)** |  |  |  |  |
| non-Hispanic Black women (N = 4,177) | 99 (1.10) | 13 | 82 (59, 118) | 842 |
| Entire NHANES Sample (N = 41,169) | 149 (1.12) | 9 | 116 (78, 177) | 6057 |
| ^1^ Hemoglobin A1c (glycohemoglobin), is a diabetes test that reflects plasma glucose for the previous 120 days. | | | | |
