# Supplementary material for: Examining educational attainment and allostatic load in non-Hispanic Black women
Source: BMC Womens Health. 2022 Mar 17;22:75. doi: 10.1186/s12905-022-01641-0 (PMC8928016; doi:10.1186/s12905-022-01641-0)
Supplement: Supplementary file 2 — Additional file 2: Supplemental Table 2. Association Between Education and High Allostatic Load Stratified by Age Groups, Presented as Prevalence Ratios (PRs) and Associated 95% Confidence Intervals (CIs), among 4,177 participants, using NHANES weighting an estimated 9,494,904 US Black Women. [file 12905_2022_1641_MOESM2_ESM.docx]

| Supplemental Table 2. Association Between Education and High Allostatic Load Stratified by Age Groups, Presented as Prevalence Ratios (PRs)^1^ and Associated 95% Confidence Intervals (CIs), among 4,177 participants, using NHANES weighting an estimated 9,494,904 US Black Women. | | | | |
| --- | --- | --- | --- | --- |
|  |  | **Prevalence Ratios (95% Confidence Interval)** | |  |
|  |  |  |  |  |
|  | **No. (Weighted %)^2^** | **Crude** | **Model 1^3^** | **Model 2^4^** |
|  | **Among women aged 18 – 29 years (*n* = 1,030)** | | | |
| **Education Level** |  |  |  |  |
| Less than High School (Referent) | 23 (13.7) | 1.000 (Referent) | 1.000 (Referent) | 1.000 (Referent) |
| High School/GED | 47 (35.8) | 1.552 (1.535 – 1.570) | 1.486 (1.464 – 1.509) | 1.250 (1.230 – 1.271) |
| Some College or Associates Degree | 44 (41.3) | 1.413 (1.398 – 1.428) | 1.656 (1.633 – 1.680) | 1.385 (1.363 – 1.407) |
| College Graduate or Higher | 10 (9.3) | 1.141 (1.124 – 1.158) | 1.152 (1.124 – 1.180) | 0.641 (0.623 – 0.660) |
|  | **Among women aged 30 – 39 years (*n* = 643)** | | | |
| **Education Level** |  |  |  |  |
| Less than High School (Referent) | 43 (22.7) | 1.000 (Referent) | 1.000 (Referent) | 1.000 (Referent) |
| High School/GED | 32 (17.7) | 0.673 (0.668 – 0.679) | 0.591 (0.585 – 0.597) | 0.657 (0.650 – 0.664) |
| Some College or Associates Degree | 77 (43.4) | 0.943 (0.936 – 0.949) | 0.964 (0.956 – 0.971) | 0.914 (0.906 – 0.922) |
| College Graduate or Higher | 27 (16.2) | 0.626 (0.621 – 0.632) | 0.610 (0.603 – 0.617) | 0.605 (0.598 – 0.612) |
|  | **Among women aged 40 – 49 years (*n* = 748)** | | | |
| **Education Level** |  |  |  |  |
| Less than High School (Referent) | 61 (19.4) | 1.000 (Referent) | 1.000 (Referent) | 1.000 (Referent) |
| High School/GED | 69 (22.2) | 1.057 (1.050 – 1.064) | 1.064 (1.056 – 1.072) | 0.986 (0.976 – 0.996) |
| Some College or Associates Degree | 132 (40.9) | 0.951 (0.945 – 0.956) | 1.018 (1.011 – 1.025) | 1.107 (1.097 – 1.117) |
| College Graduate or Higher | 53 (17.3) | 0.659 (0.634 – 0.663) | 0.735 (0.729 – 0.741) | 0.768 (0.760 – 0.777) |
|  | **Among women aged 50+ years (*n* = 1,756)** | | | |
| **Education Level** |  |  |  |  |
| Less than High School (Referent) | 316 (30.6) | 1.000 (Referent) | 1.000 (Referent) | 1.000 (Referent) |
| High School/GED | 245 (24.7) | 0.932 (0.928 – 0.935) | 0.953 (0.949 – 0.957) | 0.946 (0.942 – 0.951) |
| Some College or Associates Degree | 293 (29.5) | 0.927 (0.924 – 0.931) | 0.968 (0.964 – 0.972) | 0.973 (0.968 – 0.977) |
| College Graduate or Higher | 136 (15.2) | 0.842 (0.838 – 0.846) | 0.968 (0.963 – 0.973) | 1.021 (1.014 – 1.027) |
| ^1^ Prevalence Ratios for high allostatic load are estimated using modified Poisson regression with robust variance estimation and accounting for NHANES weighting. Confidence intervals estimated using delete-1 jackknife method accounting for complex statistical weighting, cluster, and strata.  ^2^ Number of participants with high allostatic load per stratum (weighted stratum proportion with high allostatic load).  ^3^ Model 1: Additionally adjusted for total number of pregnancies, age at menarche, and poverty to income ratio,  ^4^ Model 2: Additionally adjusted for depressive disorder, smoker status, ever congestive heart failure, and ever heart attack. | | | | |
